# Effect of W Addition on Fe-P-C-B Soft-Magnetic Amorphous Alloy

**DOI:** 10.3390/ma15238416

**Published:** 2022-11-25

**Authors:** Cheng Sun, Hongjie Xu, Yang Meng, Xinchao Wang, Minhao Han, Boying Qiao, Yalong Wang, Tao Zhang

**Affiliations:** 1 Key Laboratory of Aerospace Materials and Performance (Ministry of Education), School of Materials Science and Engineering, Beihang University, Beijing 100191, China; 2 Key Laboratory of Advanced Materials Processing Technology (Ministry of Education), School of Materials Science and Engineering, Tsinghua University, Beijing 100084, China; 3 Center for Advanced Analysis and Computational Science, Zhengzhou University, Zhengzhou 450001, China

**Keywords:** amorphous alloys, Tungsten addition, soft magnetic property, ab initio molecular dynamics simulation

## Abstract

In this work, the thermal behavior, soft magnetic properties, and structure of Fe_86−x_P_11_C_2_B_1_W_x_ (x = 0, 0.1, 0.2, 0.4, 0.6, 0.8, 1, 2, and 4) amorphous alloys were researched by several experimental methods and ab initio molecular dynamics. The addition of W improved the thermal stability of the alloy system when the first onset crystallization temperature (*T*_x1_) increased from 655 K to 711 K, significantly reduced the coercivity *H*_c,_ and decreased the saturation magnetization *B*_s_. The Fe_85.6_P_11_C_2_B_1_W_0.4_ alloy showed optimal soft magnetic performance, with low *H*_c_ of 1.4 A/m and relatively good *B*_s_ of 1.52 T. The simulation results suggested that W atoms increased the distance of the neighboring Fe-Fe pair, reduced the coordination number, narrowed the gap between the spin-up and spin-down electrons of each atom, and decreased the average magnetic moment of the Fe atoms. This work demonstrates a micro-alloying strategy to greatly reduce *H*_c_ while maintaining high *B*_s_.

## 1. Introduction

Soft magnetic materials are essential for increasing the efficiency of electrical and electronic equipment, such as transformers, memories, magnetic shields, and sensors [1,2]. Thereinto, Fe-based amorphous alloys have drawn much attention as a result of their outstanding soft magnetic performances, such as excellent saturation magnetization (*B*_s_), high effective permeability, extremely low coercivity (*H*_c_), and low core loss [3,4]. Fe-Si-B amorphous alloys are typically representative, with broad practical applications in transformers and inductors [5]. The newly developed Fe-P-C-B amorphous alloys have drawn more attention because of their superior comprehensive properties, including excellent soft magnetic properties, large electric resistivity, good processibility, and low cost [6,7,8,9]. For the green development of the next-generation devices, soft magnetic amorphous materials with lower *H*_c_ will play a significant role.

The chemical composition design has been widely applied in Fe-based amorphous alloys to improve the performance of glass-forming ability (GFA) and various properties. For this reason, multiple elements have been adopted to enhance *B*_s_ and decrease *H*_c_ values [10,11,12]. Typically, the addition of high-melting-point metals with large atoms, such as Mo, Nb, and W, can significantly affect the microstructure of the melt-spun alloys, which is helpful for obtaining good soft magnetic properties [13,14,15]. Tungsten, which shows the highest melting point among all metals, can strengthen thermal stability, extend the supercooled liquid region, and enhance the GFA of Fe-based amorphous alloys [16,17,18,19]. Moreover, W alloying can enhance the soft magnetic performances of Fe-based metallic glass and amorphous alloys. For example, the addition of the W element in Fe-Y-B amorphous alloys leads to low *H*_c_ below 2 A/m [20]. With the addition of W, Fe_61_Co_10_Zr_5_W_4_B_20_ showed a very low *H*_c_ of 1.4 A/m [21]. However, the influences of the W element on the Fe-P-based amorphous alloys, especially in newly developed Fe-P-C-B alloy systems, is unclear and has not been systematically studied.

In this work, the authors have attempted to investigate the soft magnetic performances and structure of the newly developed Fe-P-C-B amorphous alloys concerning W addition. The results suggest that the *H*_c_ of the Fe-P-C-B amorphous alloy decrease obviously with the minor addition of W. Moreover, the Vienna ab initio simulation package (VASP) was used to investigate the evolution of atomic and electronic structures caused by the composition adjustment.

## 2. Experimental

Alloys with nominal atomic compositions of Fe_86_P_11_C_2_B_1_W_x_ (x = 0, 0.1, 0.2, 0.4, 0.6, 0.8, 1, 2, and 4) were prepared by the induction-melting method in an Ar atmosphere. The specific crude materials were as follows: Fe (99.9 wt.%), C (99.9 wt.%), W (99.95 wt.%), Fe-B prealloy (B: 20.0 wt.%), and Fe-P prealloy (P: 22.1 wt.%). The ingots were remelted many times to guarantee the uniformity of composition. Amorphous ribbons with a width of approximately 1.5 mm and a thickness of approximately 20 µm were produced by single roller melt-spinning method in an Ar atmosphere. The corresponding samples were annealed at different temperatures in evacuated and sealed quartz tubes in a muffle furnace (TKD-1400, Beijing, China) to obtain inner-stress relief samples. The annealing processes of ribbons were performed in the presence of Earth’s field, and no extra external field was applied during the annealing process.

D/max-2500 PC Rigaku X-ray diffractometer (XRD, D/MAX 2500pc, Tokyo, Japan) with Cu Kα radiation was used to investigate the amorphous nature of the melt-spun ribbons and the microstructure of annealed samples. The scanning range was from 30° to 90°, and the scanning rate was 2 degrees per minute. Thermodynamic parameters of melt-spun ribbons were identified by using a differential scanning calorimetry (DSC, PerkinElmer DSC 8000, Waltham, MA, USA) under an Ar gas flow with a heating rate of 0.33 K/s. The Archimedes principle has been used to measure the density of alloys at normal atmospheric temperatures. The hysteresis loops identified the *B*_s_ of the melt-spun and annealed ribbons, which were measured by Lakeshore 7407 vibrating sample magnetometer (VSM, Lake Shore 7407, Westerville, OH, USA) under an applied field of 800 kA/m. A DC B-H loop tracer (MATS-2010SA, Loudi, China) was used to measure the *H*_c_ of the ribbons under a field of 800 A/m. Moreover, the bright field and the high-resolution transmission electron microscope (HRTEM) microstructure of the samples were characterized via TEM (JEM-2100 F, Tokyo, Japan). The TEM samples were prepared by ion thinning. The magnetic domain structure was observed by the magneto-optical Kerr effect microscope.

By means of density functional theory, the structure of Fe_86_P_11_C_2_B_1_, Fe_85.5_P_11_C_2_B_1_W_0.5,_ and Fe_85_P_11_C_2_B_1_W_1_ amorphous alloys was simulated by using the Vienna Ab initio simulation package (VASP) [22]. The projected augmented wave method was used to describe electron-ion interactions [23]. Exchange-correlation potentials were calculated by Perdew–Burke–Ernzerh mode with a generalized gradient approximation [24]. The primitive volume of the cubic periodic supercell containing 200 atoms was decided by the experimental density of Fe_86_P_11_C_2_B_1_, Fe_85.5_P_11_C_2_B_1_W_0.5,_ and Fe_85_P_11_C_2_B_1_W_1_ amorphous alloys at a normal atmospheric temperature. The systems were melted and well equilibrated for 6000 steps (2 fs for each step) at 2000 K and subsequently quenched to 300 K at a cooling rate of 5 × 10^14^ K/s. In the end, the systems were equilibrated for 5000 steps at 300 K, and at least 1000 configurations were collected to analyze the structure.

## 3. Results and Discussion

### 3.1. Amorphous Nature and Thermal Behavior

**Figure 1:**
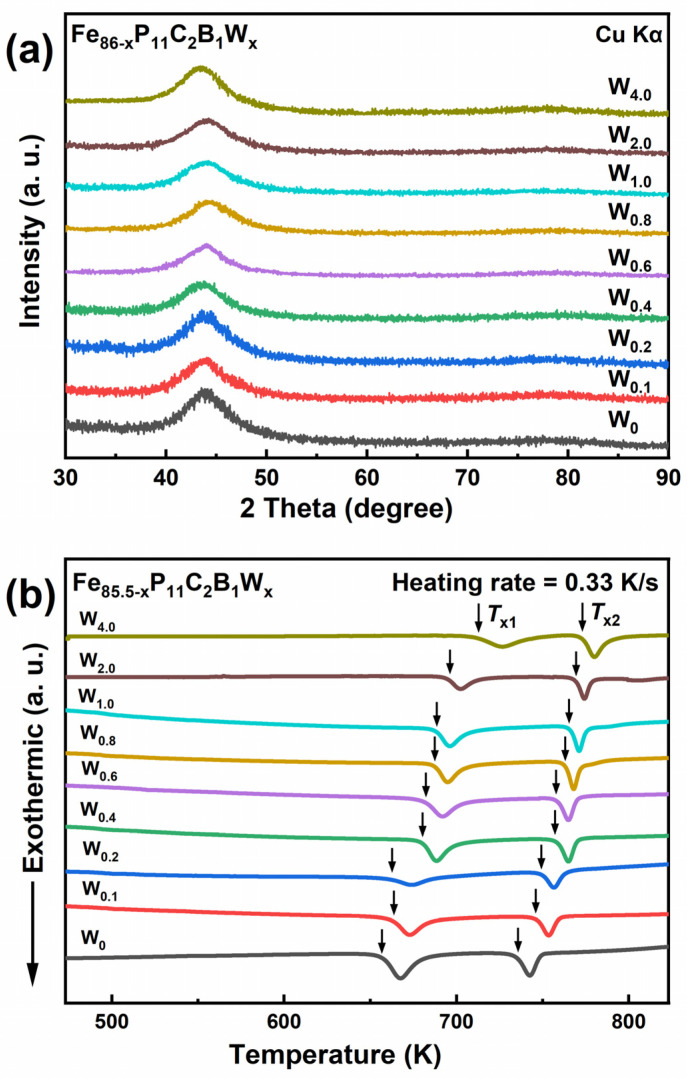
(**a**) XRD patterns and (**b**) DSC curves of the melt-spun Fe_86−x_P_11_C_2_B_1_W_x_ ribbons.

In order to identify the optimal content of the W element in the Fe-P based amorphous alloys, Fe_86−x_P_11_C_2_B_1_W_x_ (x = 0, 0.1, 0.2, 0.4, 0.6, 0.8, 1, 2, and 4) (also denoted as W_x_ hereafter) ribbon samples were prepared. Figure 1a exhibits XRD patterns of all melt-spun ribbons with different proportions of W elements. All patterns show broad humps without undetectable crystalline features at the 2*θ* of about 45°, which indicates the amorphous nature of these samples.

As shown in Figure 1b, the crystallization of the melt-spun Fe_86−x_P_11_C_2_B_1_W_x_ (x = 0, 0.1, 0.2, 0.4, 0.6, 0.8, 1, 2, and 4) amorphous alloys was investigated by DSC with a heating rate of 0.33 K/s. Each DSC curve exhibits two exothermic peaks with similar heat release, indicating a two-step crystallization process for these samples. It was reported that the first exothermic peak relates to the precipitation of the soft magnetic phase (α-Fe), while the second peak relates to the hard magnetic phase [25]. No distinct glass transition phenomenon can be observed for all the samples. The relevant thermal parameters, including the first crystallization onset temperature (*T*_x1_), the second crystallization onset temperature (*T*_x2_), and the temperature interval Δ*T* (Δ*T* = *T*_x2_ − *T*_x1_), are listed in Table 1. Moreover, it can be seen that *T*_x1_ (from 655K to 711 K) and *T*_x2_ (from 736 K to 773 K) rise with the addition of the W element. This shows that the content of W significantly affects the thermostability of the amorphous phase. Considering the Δ*H*_mix_ (enthalpy of mixing) of W-P, W-C, and W-B are −46.5 kJ/mol, −60 kJ/mol, and −31 kJ/mol, respectively [26], it is not surprising because the W atom can strengthen the cohesive energy through the formation of W-metalloid bonds within Fe-based amorphous alloys, which reduce the diffusion ability of metalloid elements in the amorphous structure and impede the precipitation of α-Fe from the amorphous phase [27].

### 3.2. Magnetic Properties and Microstructure Analysis

**Figure 2:**
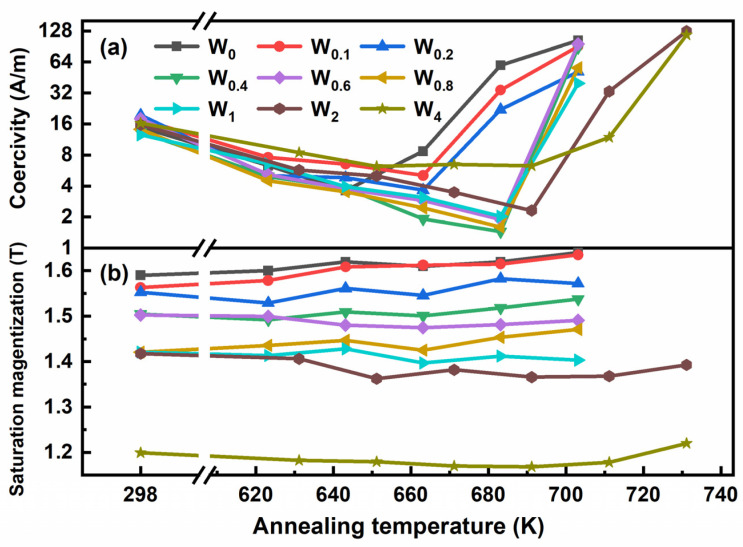
Annealing temperatures dependence of (**a**) *H*_c_ and (**b**) *B*_s_ for Fe_86−x_P_11_C_2_B_1_W_x_ alloy ribbons.

For magnetic Fe-based amorphous alloys, the soft magnetic performances can be enhanced after annealing at the proper temperature [28,29]. All samples were annealed at different annealing temperatures (*T*_a_) for 10 min. After the annealing process, the magnetic performance and corresponding microstructure of Fe_86−x_P_11_C_2_B_1_W_x_ alloys were analyzed. All *H*_c_ curves exhibit V-shaped features, as shown in Figure 2a, which shows low *H*_c_ for *T*_a_ ≤ *T*_x1_ and two *H*_c_ maxima at low and high temperature ends. The high *H*_c_ of the melt-spun ribbons is considered to originate from the internal stress caused by rapid solidification during the melt-spinning process, which can be reduced by a proper annealing process [30]. When *T*_a_ is higher than *T*_x1_, *H*_c_ increases rapidly when *T*_a_ is higher than *T*_x1_, as a result of the rapid growth of α-Fe grains and the precipitation of Fe_3_(P, C, B) compounds [31]. The appropriate addition of the W element can reduce the *H*_c_ values of Fe_86−x_P_11_C_2_B_1_W_x_ alloys. According to previous research, structural defects and surface irregularities have a great influence on *H*_c_ [32,33,34]. The addition of W may reduce the structural defects and surface irregularities of FePCB amorphous alloys, which lead to the reduction in *H*_c_ values. The lowest *H*_c_ value and corresponding *T*_a_ and *B*_s_ values for each alloy are listed in Table 2. Compared to the original Fe_86_P_11_C_2_B_1_ alloy, W_x_ (≤2 at.%) alloys show lower *H*_c_ values after a proper annealing process. Specifically, the W_0.4_, W_0.6,_ and W_0.8_ alloys exhibit relatively low *H*_c_ of 1.4 A/m, 1.9 A/m, and 1.6 A/m, respectively, when *T*_a_ is around *T*_x_. In addition, the *B*_s_ of all alloys was also investigated at different annealing temperatures, as shown in Figure 2b. The *B*_s_ values monotonically decrease with the increase in W addition at most temperatures. These characteristics can be explained by the reduction in Fe content and the distance increase between Fe-Fe atoms caused by W addition [35].

**Figure 3:**
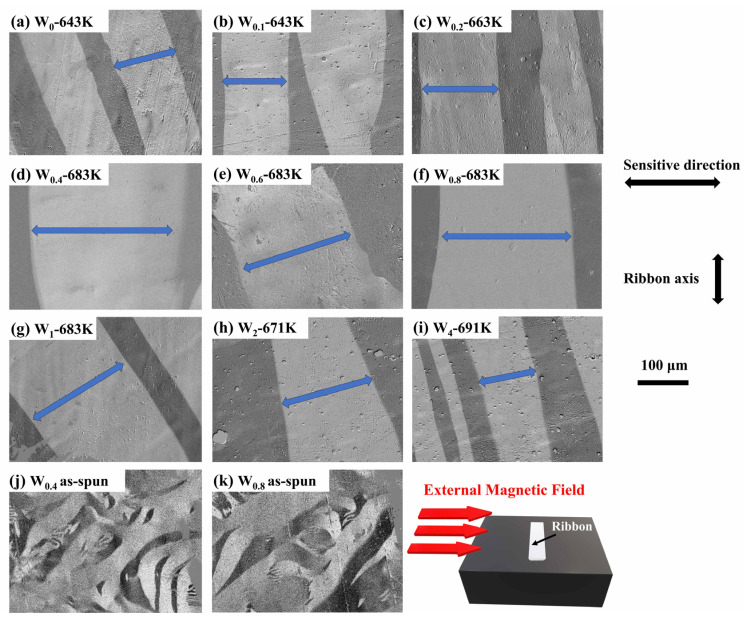
Magnetic domains of (**a**,**b**) W_0_ and W_0.1_ ribbon annealed at 643 K; (**c**) W_0.2_ ribbon annealed at 663 K; (**d**–**g**) W_0.4_, W_0.6_, W_0.8_ and W_1_ ribbon annealed at 683 K; (**h**) W_2_ ribbon annealed at 671 K; (**i**) W_4_ ribbon annealed at 691 K; (**j**,**k**) as-spun W_0.4_ and W_0.8_ ribbon. The annealing time is 10 min. The magnitude of the field is 20 Oe, and the orientation of this field is perpendicular to the direction of the long axis of the ribbon.

Considering the relationships between magnetic domain structure and magnetic property, the MOKE microscopy characterization of different Fe_86−x_P_11_C_2_B_1_W_x_ ribbons was carried out. Table 2 lists the specific composition and related annealing temperature. The annealing time is 10 min. As can be seen from Figure 3a–i, the wide straight strip domains with smooth edges can be observed, which indicates a homogeneous magnetic structure, a low pinning effect, and small anisotropy of the annealed ribbons. Some defects in domains can be attributed to the edge effect, which is mainly induced by the surface quality and change of inner-stress direction [36]. In contrast, mazing fingerprint-like domains can be seen in Figure 3j,k. The formation of maze domains is mainly due to the non-uniformity internal stress induced by rapid solidification during the preparation of amorphous alloys [37]. The near-field image also shows the sensitive direction representing the vibration direction of incident polarized light and the strip axis. Accordingly, the results provide solid evidence for the low *H*_c_ of all annealed ribbons, especially for W_0.4_, W_0.6,_ and W_0.8_ ribbons. The lower domain wall energy $\gamma_{B}$ causes the widening of the domain, which is correlated to the weak pinning effects induced by the anisotropy fluctuations of the materials and internal stress [38].

**Figure 4:**
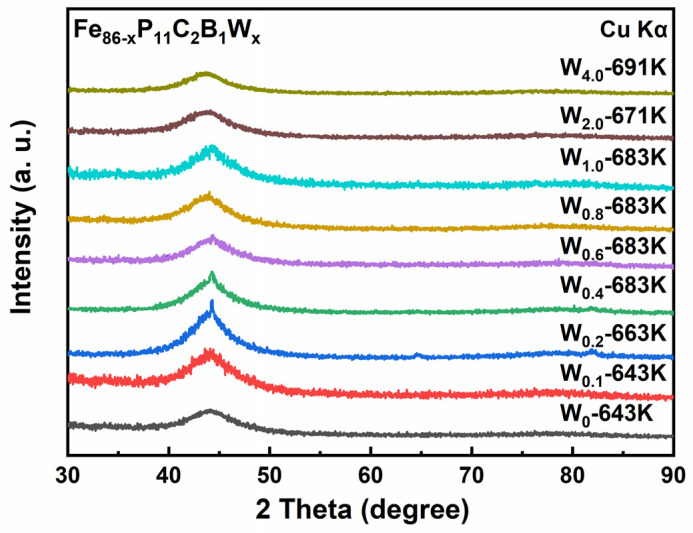
XRD patterns of the annealed Fe_86−x_P_11_C_2_B_1_W_x_ ribbons.

**Figure 5:**
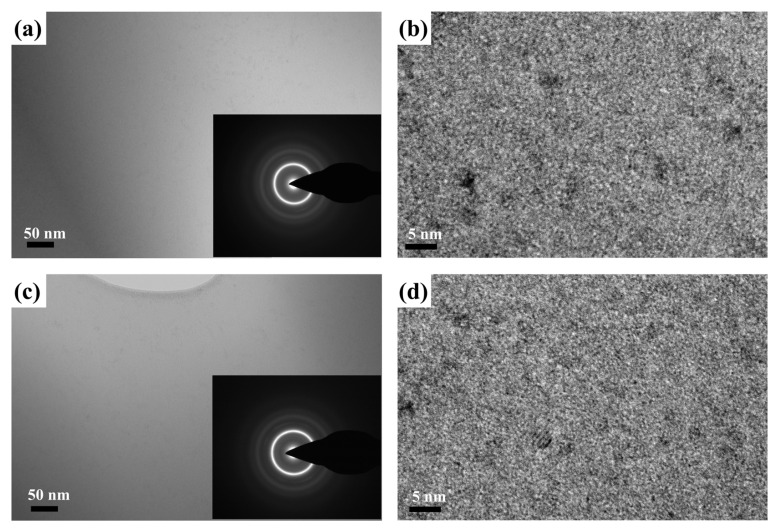
(**a**) Bright-field TEM and (**b**) HRTEM pictures of Fe_85.6_P_11_C_2_B_1_W_0.4_ sample annealed at 683 K for 10 min. (**c**) Bright-field TEM and (**d**) HRTEM pictures of Fe_85.6_P_11_C_2_B_1_W_0.8_ sample annealed at 683 K for 10 min. The insets in (**a**,**c**) are the corresponding SAED patterns.

Figure 4 presents the XRD images of annealed samples listed in Table 2. Crystalline peaks can be clearly seen from the W_0.2_ and W_0.4_ ribbons, and minor peaks can be observed from W_0.6_ and W_0.8_ XRD patterns. According to G. Herzer’s effective magnetic random anisotropy model, the precipitation of fine and regular nanocrystalline can reduce the *H*_c_ of amorphous alloys [39]. This helps explain why W_x_ (x = 0.2, 0.4, 0.6, 0.8, and 1) ribbons show the lowest *H*_c_ when *T*_a_ is around *T*_x_. Furthermore, TEM is adopted to investigate the phase structure of the annealed Fe_85.6_P_11_C_2_B_1_W_0.4_ and Fe_85.2_P_11_C_2_B_1_W_0.8_ alloy ribbons (683 K for 10 min). As exhibited in Figure 5a,c, the bright field TEM image shows the primary amorphous phase for the annealed ribbons, as also confirmed by the inset selected area electron diffraction (SAED) pattern. As shown in Figure 5b,d, crystallographic planes indicating the presence of nanocrystals can be seen in HRTEM images, which is consistent with the XRD results.

### 3.3. Simulation of Amorphous Structure and Magnetic Properties

**Figure 6:**
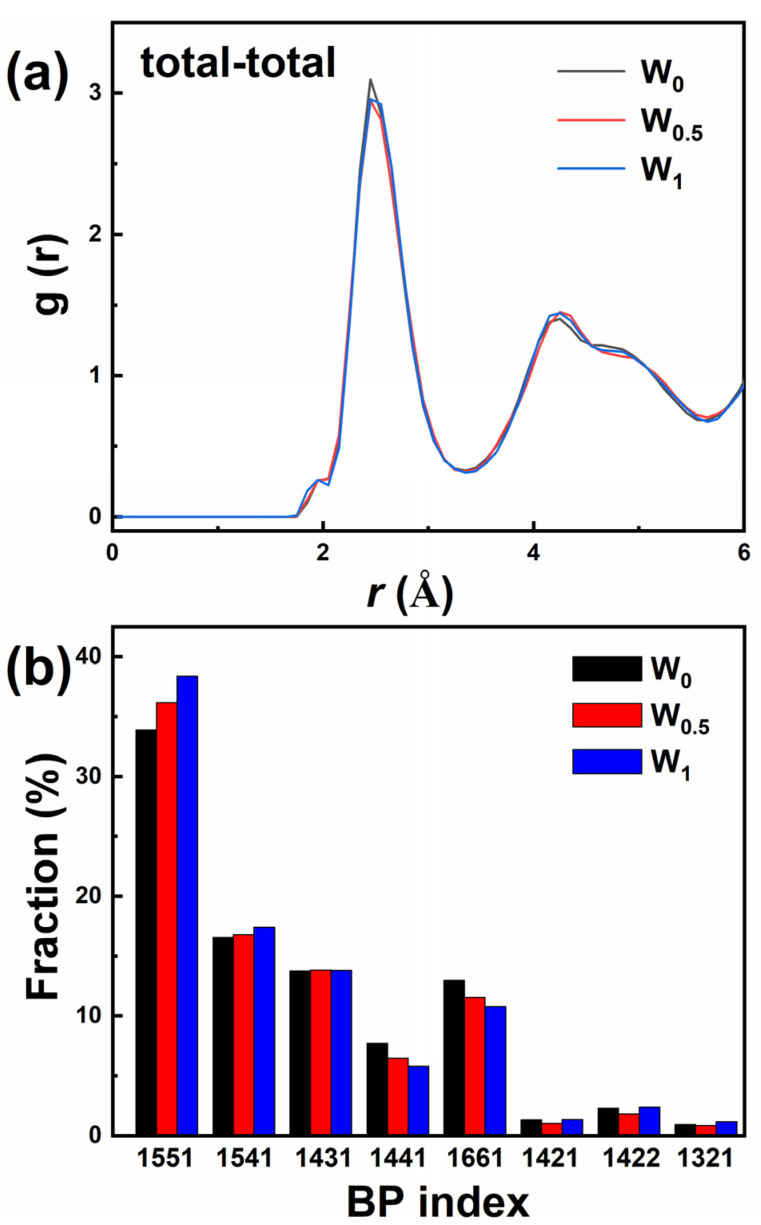
(**a**) Pair correlation functions and (**b**) bond pairs distribution of Fe_86−x_P_11_C_2_B_1_W_x_ (x = 0, 0.5, and 1) amorphous alloys.

To characterize the structure and magnetic performances of amorphous alloys, AIMD is an effective approach. According to the experimental results, the minor addition of the W element causes large changes in magnetic properties. Therefore, Fe_86_P_11_C_2_B_1_, Fe_85.5_P_11_C_2_B_1_W_0.5,_ and Fe_85_P_11_C_2_B_1_W_1_ are selected as representative alloy components for comparison. The pair correlation function (PCF) of Fe_86−x_P_11_C_2_B_1_W_x_ (x = 0, 0.5 and 1) amorphous alloys at a normal atmospheric temperature (300 K) is shown in Figure 6a. The prominent first peak indicates the characteristics of short-range order, while the second one with splitting feature indicates mid-range order, which indicates the amorphous nature of Fe_86−x_P_11_C_2_B_1_W_x_ (x = 0, 0.5 and 1) alloys. Moreover, the relative strength of the first peak shows a slight decrease, and the second peak increases after adding the W element. This indicates that the adjacent structure of the atoms is changed after W addition. Figure 6b exhibits the distribution of bond pairs (BPs) of the Fe_86−x_P_11_C_2_B_1_W_x_ (x = 0, 0.5, and 1) amorphous alloys. The BPs analysis proposed by previous researchers is an effective method for describing the local atomic structure [40]. The BPs between the central and nearest-neighbor atoms are characterized by the first valley of the related partial PCF curve. 1551, 1541, and 1431 BPs represent icosahedral order, while the 1441 and 1661 BPs are features of the body-centered cubic (bcc) structures [41]. The percentage of icosahedral clusters is positively related to the enhanced GFA [42]. With the addition of W, the proportion of icosahedral BPs increases, while the proportion of bcc crystalline BPs decreases significantly. The results indicate that W can enhance the GFA of Fe_86−x_P_11_C_2_B_1_W_x_ amorphous alloys, which is in agreement with previous research [18,19,20,27].

**Figure 7:**
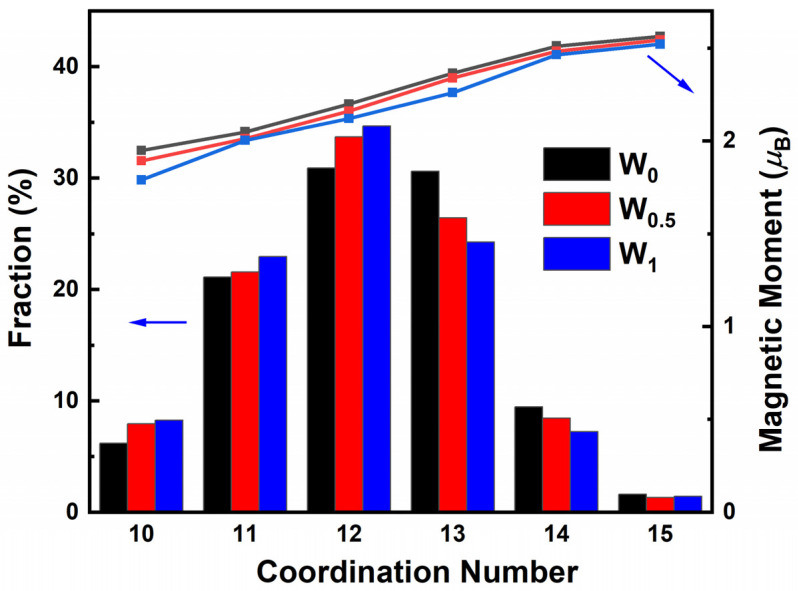
Fe magnetic moment distribution with corresponding coordination number in the Fe_86−x_P_11_C_2_B_1_W_x_ (x = 0, 0.5, and 1) amorphous alloys.

To further understand the effects of W addition on soft magnetic performances, atomic magnetic moments were analyzed. The average magnetic moments of the Fe, P, C, B, and W atoms were calculated by AIMD for the Fe_86−x_P_11_C_2_B_1_W_x_ (x = 0, 0.5 and 1) amorphous alloys. As summarized in Table 3, the electronic magnetic moment of the Fe atoms dominates the total magnetic moments of the whole alloy system. The average magnetic moment of the Fe atoms decreases from 2.225 *μ*_B_ to 2.096 *μ*_B_ with the addition of the W element. In addition, W exhibits a more negative magnetic moment (−0.578 *μ*_B_) than other constituent elements. This can explain the previous experimental phenomenon that the *B*_s_ values monotonically decrease with the addition of W. Furthermore, the Fe magnetic moment distribution with the corresponding coordination number was concerned. The average magnetic moment of Fe in Table 3 can be calculated from the weighted average of the data in Figure 7. The coordination number is defined as the number of the first nearest neighbor Fe atoms near the central Fe atoms. The proportion of the high coordination number in the first nearest neighbor was reported to correlate positively with the magnetic moment [43]. This is because the increase in the proportion of high coordination numbers in the first nearest neighbor indicates more neighboring Fe atoms taking part in the ferromagnetic coupling, which increases saturated magnetization [44]. It can be seen in Figure 7 that the proportion of high coordination numbers (13, 14) decreases. In contrast, the proportion of low coordination numbers (10, 11, and 12) increases with the addition of W. This result shows that the addition of W expands the nearest neighbor Fe-Fe spacing and diminishes the percentage of high coordination numbers. Thus, the average magnetic moment of Fe atoms decreases, which leads to a decrease in the *B*_s_ of the Fe_86−x_P_11_C_2_B_1_W_x_ (x = 0, 0.5 and 1) amorphous alloys.

**Figure 8:**
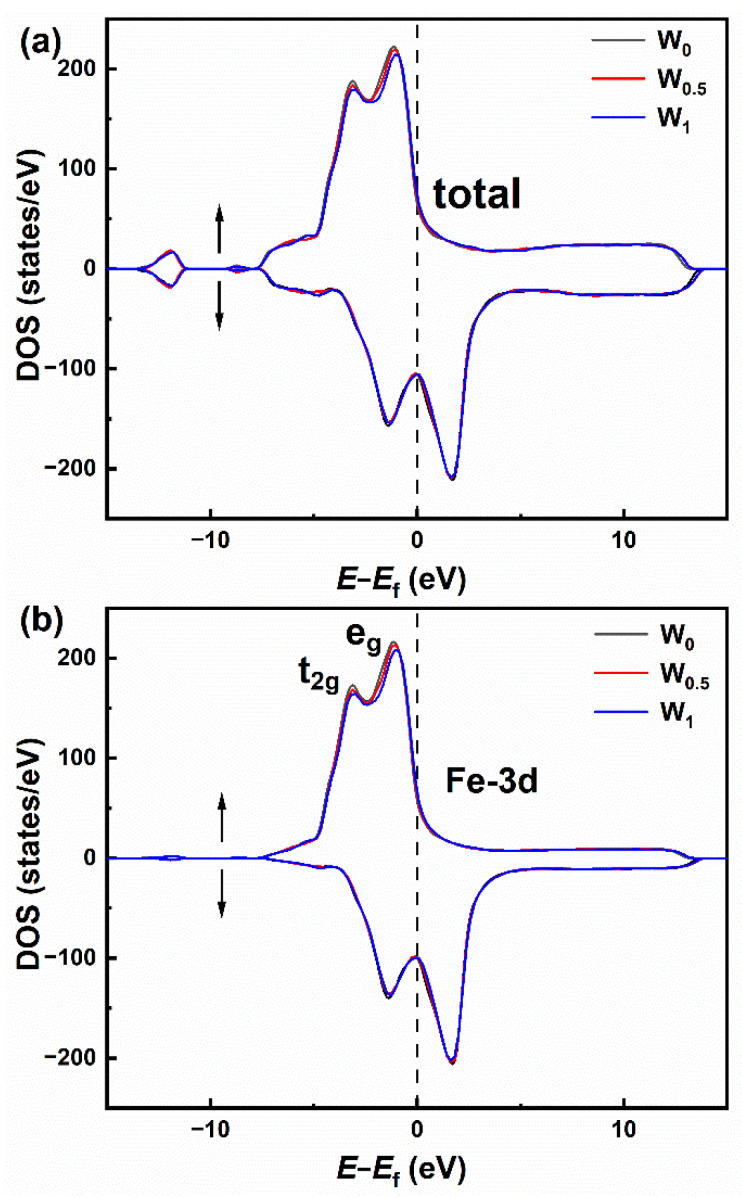
(**a**) Total electron DOS and (**b**) partial electron DOS of Fe atoms (3d orbital) in the Fe_86−x_P_11_C_2_B_1_W_x_ (x = 0, 0.5 and 1) amorphous alloys. The arrows indicate the spin direction of the electron.

The tunable electronic performances of Fe_86−x_P_11_C_2_B_1_W_x_ (x = 0, 0.5 and 1) alloys were also researched by the density of states (DOS). The total electron DOS of the Fe atom in Fe_86−x_P_11_C_2_B_1_W_x_ (x = 0, 0.5, and 1) amorphous alloy is illustrated in Figure 8a. The Fermi level lay right above the majority spin band and close to the minimum value of the spin-down band, indicating that ferromagnetism exists in the alloy [45]. Figure 8b shows the partial electron DOS (3d orbital) of Fe atoms for Fe_86−x_P_11_C_2_B_1_W_x_ (x = 0, 0.5 and 1) amorphous alloys. Two dominant peaks separated from the 3d band states can be seen, which were reported as the lower-energy t_2g_ orbital and the higher-energy e_g_ orbital [46]. According to previous research, ferromagnetism is caused by the distinction in the magnetic moments of the upper and lower spin electrons induced by the asymmetry of band splitting [47]. With the addition of the W element, the number of spin-up electrons N↑ decreases slightly, while the number of occupied spin-down electrons N↓ is almost unchanged. Therefore, the gap becomes much closer, which leads to a decreased Fe atomic magnetic moment of the Fe atom in the alloy system.

## 4. Conclusions

Fe_86−x_P_11_C_2_B_1_W_x_ (x = 0, 0.1, 0.2, 0.4, 0.6, 0.8, 1, 2, and 4) amorphous alloys were developed by melt spinning, and the influences of W addition on the amorphous structure, thermal behavior, and soft magnetic performances were researched by several experiments and AIMD simulation. The addition of W enhances the thermal stability, with *T*_x1_ and *T*_x2_ increasing up to 711 K and 731 K, respectively, consistent with the BP analysis of the simulation, which suggests that the GFA of the amorphous alloy can be improved with W addition. The amorphous alloy systems exhibit good *B*_s_ and low *H*_c_ in the range of 1.17–1.62 T and 1.4–6.3 A/m. The low *H*_c_ of annealed ribbons was proved by straight wide stripy magnetic domains. Especially, the Fe_85.6_P_11_C_2_B_1_W_0.4_ alloy shows optimal soft magnetic performances, with low *H*_c_ of 1.4 A/m and relatively good *B*_s_ of 1.52 T. The magnetic moment and electron DOS analysis demonstrates that the decrease in *B*_s_ via W addition results from the distance increase in nearest neighboring Fe-Fe, gap narrowing between spin-up N↑ and spin-down N↓ electrons, diminishment of the coordination number, and a reduction in the average magnetic moment of the Fe atoms.

## Figures and Tables

**Table 1:** Thermal parameters of Fe_86−x_P_11_C_2_B_1_W_x_ amorphous alloys.

Alloys	Thermal Parameters		
	*T*_x1_ (K)	*T*_x2_ (K)	Δ*T* (K)
Fe_86_P_11_C_2_B_1_	655	736	81
Fe_85.9_P_11_C_2_B_1_W_0.1_	661	746	84
Fe_85.8_P_11_C_2_B_1_W_0.2_	660	749	89
Fe_85.6_P_11_C_2_B_1_W_0.4_	680	758	78
Fe_85.4_P_11_C_2_B_1_W_0.6_	682	760	78
Fe_85.2_P_11_C_2_B_1_W_0.8_	687	763	76
Fe_85_P_11_C_2_B_1_W_1_	688	766	78
Fe_84_P_11_C_2_B_1_W_2_	692	768	76
Fe_82_P_11_C_2_B_1_W_4_	711	773	62

**Table 2:** The *H*_c_ and *B*_s_ values of Fe_86−x_P_11_C_2_B_1_W_x_ alloys annealed at optimal temperature for 10 min.

Alloys	*T*_a_ (K)	*H*_c_ (A/m)	*B*_s_ (T)
Fe_86_P_11_C_2_B_1_	643	3.6	1.62
Fe_85.9_P_11_C_2_B_1_W_0.1_	643	3.5	1.61
Fe_85.8_P_11_C_2_B_1_W_0.2_	663	3.7	1.55
Fe_85.6_P_11_C_2_B_1_W_0.4_	683	1.4	1.52
Fe_85.4_P_11_C_2_B_1_W_0.6_	683	1.9	1.48
Fe_85.2_P_11_C_2_B_1_W_0.8_	683	1.6	1.45
Fe_85_P_11_C_2_B_1_W_1_	683	2.0	1.41
Fe_84_P_11_C_2_B_1_W_2_	671	2.3	1.38
Fe_82_P_11_C_2_B_1_W_4_	691	6.3	1.17

**Table 3:** Average magnetic moments of each atomic orbital for the Fe, P, C, B, and W atoms in the Fe_86−x_P_11_C_2_B_1_W_x_ (x = 0, 0.5 and 1) amorphous alloys.

Alloys	Element	s (*μ*_B_)	p (*μ*_B_)	d (*μ*_B_)	Total (*μ*_B_)
Fe_86_P_11_C_2_B_1_	Fe	−0.009	−0.034	2.268	2.225
	P	−0.005	−0.082	0.000	−0.087
	C	−0.015	−0.126	0.000	−0.141
	B	−0.026	−0.137	0.000	−0.164
Fe_85.5_P_11_C_2_B_1_W_0.5_	Fe	−0.008	−0.035	2.250	2.207
	P	−0.006	−0.084	0.000	−0.090
	C	−0.015	−0.126	0.000	−0.141
	B	−0.027	−0.134	0.000	−0.161
	W	−0.027	−0.039	−0.521	−0.587
Fe_85_P_11_C_2_B_1_W_1_	Fe	−0.008	−0.033	2.137	2.096
	P	−0.005	−0.077	0.000	−0.082
	C	−0.013	−0.108	0.000	−0.121
	B	−0.026	−0.127	0.000	−0.153
	W	−0.027	−0.039	−0.513	−0.578

## Data Availability

The data presented in this study are available on request from the corresponding author. The data are not publicly available due to the data also forms part of an ongoing study.
